# The Impact of the COVID-19 Pandemic on Pediatric Clinical Practice in Wenzhou, China: A Retrospective Study

**DOI:** 10.3389/fped.2020.585629

**Published:** 2020-11-16

**Authors:** Hui Zhang, Li-Wei Guo, Yu-Yan Gao, Hao Yao, Zuo-Kai Xie, Wei-Xi Zhang

**Affiliations:** ^1^Department of Pediatric Allergy and Immunology, The Second Affiliated Hospital and Yuying Children's Hospital of Wenzhou Medical University, Wenzhou, China; ^2^The Second Clinical Medical College, Wenzhou Medical University, Wenzhou, China; ^3^Department of Medical Records, The Second Affiliated Hospital and Yuying Children's Hospital of Wenzhou Medical University, Wenzhou, China

**Keywords:** COVID-19, impact, pediatric, clinical practice, retrospective

## Abstract

**Introduction:** The COVID-19 pandemic has affected all aspects of life worldwide. The aim of the present study was to review and describe and acknowledge the impact of COVID-19 on the pediatric health care system at a pediatric tertiary hospital in Wenzhou.

**Methods:** A retrospective study was conducted at Yuying Children's Hospital of Wenzhou Medical University, a public pediatric tertiary hospital in Southern Zhejiang Province that specializes in pediatrics. The data regarding the primary diagnosis of patients were extracted from the electronic medical records system of the hospital. Data for outpatients and inpatients treated at the pediatric department were analyzed in the time frame of 22 weeks since the beginning of the pandemic (from December 30, 2019 to June 2, 2020) and compared with data from the same period in 2019.

**Results:** The total number of outpatient cases in the previous 22 weeks of the year declined from 560,620 in 2019 to 247,030 in 2020, and inpatient cases decreased from 14,177 to 7,555. This negative trend settled by week 6 and 7 and subsequently approached the 2019 numbers. The most noticeable decrease in the number of cases was observed in children of preschool age. Moreover, the number of weekly visits decreased at the beginning of the epidemic, reached the lowest value during the lockdown period, and recovered after the lockdown.

**Conclusion:** Based on the results of this study, clinical practice in a pediatric department in Wenzhou was substantially affected by the epidemic and measures such as physical distancing and increased personal hygiene, particularly in preschool-age children. An understanding of the trends and impacts of the pandemic on pediatric patients and health systems will facilitate better preparation of pediatricians in the future.

## Introduction

The world has seen the onset of a pandemic of the coronavirus disease 2019 (COVID-19) since December 2019 (1). Due to its extremely high potential for dissemination, the COVID-19 outbreak was declared a pandemic by the World Health Organization (2). According to WHO, COVID-19 has affected almost all countries/areas of the world and caused more than 1.1 million deaths as of October 18, 2020 (3). Patients with COVID-19 have a poor quality of life at follow-up (4).

Wenzhou, a city in Zhejiang Province with 9 million residents, has frequent travel connections with Wuhan (5). Thus, Wenzhou has faced the challenging and novel SARS-CoV-2 situation by experiencing the rapid and acute spread of the pandemic since the beginning of the outbreak, with 504 confirmed cases. Wenzhou immediately implemented preventive measures, including personal protective measures and social distancing, to contain the spread of COVID-19. Face mask wearing was enforced in public places, all enterprises and large-scale entertainment establishments were shut down, and public transportation was generally suspended. In addition to these measures, some specialized interventions were implemented for children, such as the extension of the Lunar New Year holidays, the closure of schools, and the use of distance education (6). Early responses followed by large-scale measures were proven to be effective at combating COVID-19 in our province (7). In the middle of April, the outbreak had been well-controlled in Wenzhou, and students began to return to school in batches.

The COVID-19 pandemic has dramatically affected clinical practice, resulting in an enormous challenge for health systems. The outbreak has been characterized by a substantially increased demand for respiratory and infection departments and intensive care units (ICUs). In addition, previous studies have reported a phenomenon wherein departments with limited connections with the COVID-19 situation had a reduced number of consultations (8, 9). The purpose of this study was to describe the profound changes in clinical practice that occurred in a children's hospital during the spread of the COVID-19 pandemic in Wenzhou.

## Methods

A retrospective study was conducted at Yuying Children's Hospital of Wenzhou Medical University, a public pediatric tertiary hospital in Wenzhou that specializes in pediatrics. During the COVID-19 pandemic, our hospital introduced significant adaptations to address the outbreak, including practicing pre-triage, instituting a fever clinic, and increasing tele-medicine. Seventy-two patients with a confirmed COVID-19 diagnosis, including 1 child, were treated in our hospital.

The first patient with COVID-19 in Wenzhou was hospitalized with flu-like symptoms and a Wuhan travel history on January 4, 2020 and was diagnosed on January 17, 2020. Therefore, we chose the 1st week from December 30, 2019 to January 5, 2020 as the beginning of the outbreak. We collected 22 weeks of data during the COVID-19 outbreak (from December 30, 2019 to June 2, 2020) for analysis, as well as data from the same period in 2019 (from December 31, 2018, to May 31, 2019). Furthermore, we divided the period into before lockdown (December 30, 2019, to January 31, 2020), during lockdown (February 1, 2020, to February 20, 2020), after lockdown (February 21, 2020, to April 12, 2020), and school reopening (April 13, 2020 to May 31, 2020). The data regarding the primary diagnosis of patients were extracted from the electronic medical records system of the hospital. The diagnosis of diseases was based on the International Classification of Diseases (ICD), 10th Revision. Two well-trained and experienced pediatricians (HZ and WXZ) confirmed the diagnosis. The following data were collected weekly: the numbers of patients, ages, sexes, residences, clinical departments, and major diagnoses. We selected children aged between 0 and 16 years and divided them into four groups: infants and toddlers (0–3 years), preschool age children (4–7 years), school age children (8–11 years), and adolescents (12–16 years). According to the primary diagnosis, we classified all pediatric patients as having an infectious or non-infectious disease. We calculated the rate of emergency room observations in outpatients and the rate of the intensive care unit (ICU) admissions for inpatients weekly to observe the effect of the pandemic on patients with severe cases. Patients with incomplete basic information or uncertain diagnoses were excluded.

Ethical approval was obtained from the Institutional Review Board of the Second Affiliated Hospital and Yuying Children's Hospital of Wenzhou Medical University, and the requirement for informed consent was waived because we used anonymized data.

### Statistical Analysis

All statistical analyses were conducted using the Statistical Package for the Social Sciences (SPSS) (version 23). The measured data are reported as the medians and interquartile ranges, and the Mann-Whitney U test was used for comparisons between groups. The count data are reported as frequencies, and comparisons between groups were performed using Pearson's chi-square tests. A *p* < 0.05 was considered statistically significant.

## Results

Compared with those in the same period in 2019, the numbers of outpatients and inpatients decreased significantly during the epidemic. Fewer patients were treated from January to May 2020 than in 2019: 99,388 VS 130,736, 11,388 VS 86,563, 32,626 VS 110,336, 44,258 VS 108,618, and 59,370 VS 124,367 outpatients; 810 VS 2,518, 1,140 VS 2,911, 1,442 VS 2,725, and 1,158 VS 3,052 inpatients, respectively, except January (3,005 VS 2,971).

**Figure 1:**
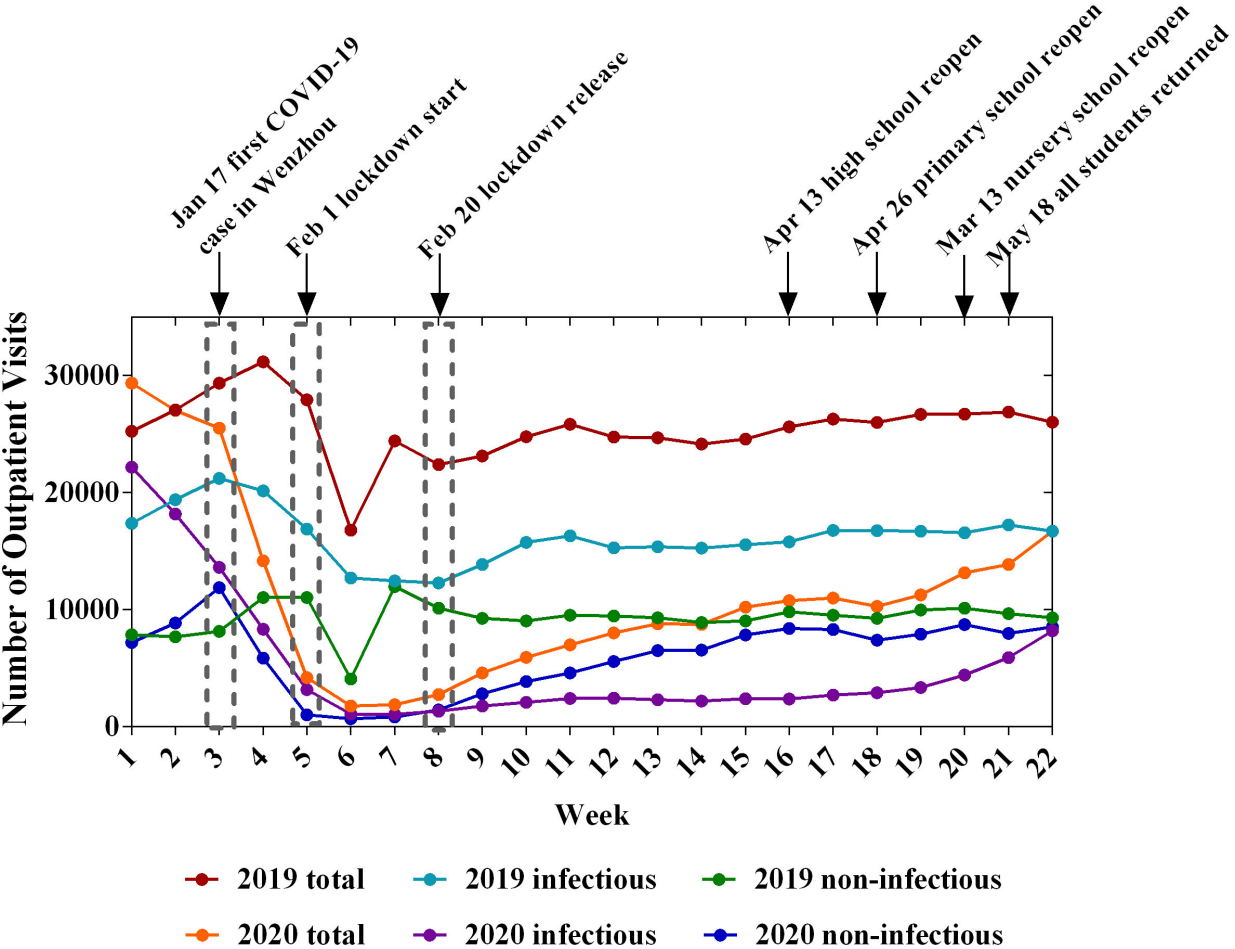
Changes of outpatient clinics during the 22-week period in 2019 and 2020.

**Figure 2:**
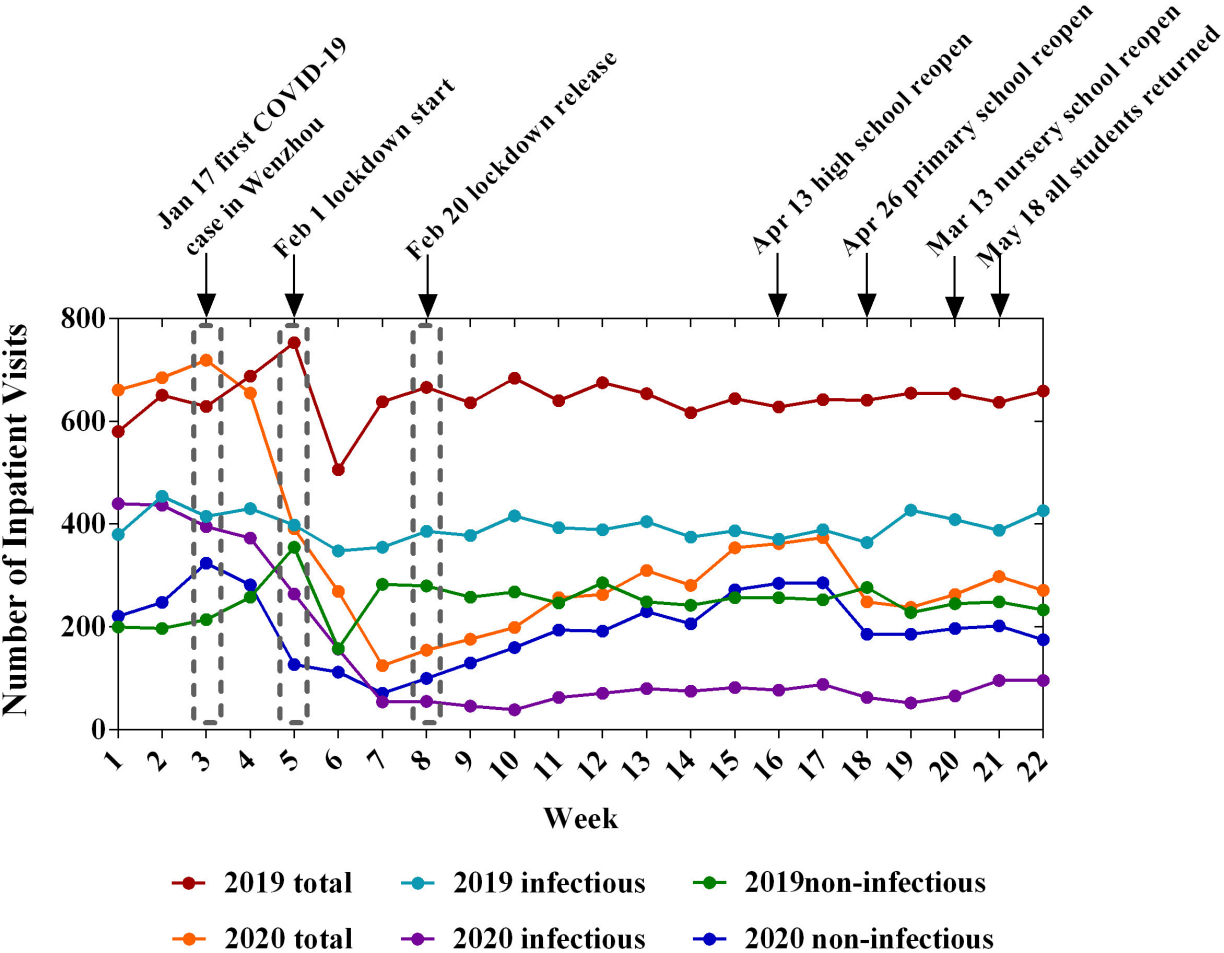
Changes of inpatient clinics during the 22-week period in 2019 and 2020.

Table 1 summarizes the characteristics of the pediatric clinical practices. The proportion of local residents of Wenzhou increased from 86.58% to 90.43% (*P* < 0.001) for outpatients. The weekly numbers of inpatients and outpatients decreased significantly during COVID-19. After the Lunar Spring Festival holiday of 2019, the number of patients recovered quickly to a high level. However, in 2020, the lowest value was observed between the 6th and 7th week and the number of patients recovered slowly. At the end of the 22nd week, the number of patients had not reached the level observed in 2019 (Figures 1, 2). The most obvious decreases in the numbers of outpatients and inpatients were in the infant and toddler group and preschool group (Table 2).

**Table 1:** The numbers of outpatients and inpatients treated by physicians in pediatric departments during the same periods in 2019 and 2020.

**Subtype^**a**^**	**Period**	**Number**	**Age, years**	**Wenzhou**	**Infectious diseases**
Outpatient	2019	560,620	4 (2–7)	86.6%	63.6%
	2020	247,030	5 (2–9)	90.4%	46.3%
Z/X^2^			−92.77	891.53	21,108.93
p^b^			0.000	0.000	0.000
Inpatient	2019	14,177	2 (0–6)	78.4%	61.2%
	2020	7,555	4 (1–8)	68.9%	41.9%
Z/X^2^			−5.71	239.92	740.54
p^b^			0.000	0.000	0.000

a Median (25th−75th percentile) for continuous variables, number for categorical variables.

b Mann-Whitney U-Test for continuous variables, Pearson Chi-squared test for categorical variables.

*A p < 0.05 was considered statistically significant*.

**Table 2:** The number of visits for different age groups in 2019 vs. 2020.

**Subtype**	**Year**	**Age groups**				**Total**	***Z***	***p***
		**Infant and toddler**	**Preschool**	**School-age**	**Adolescence**			
Outpatient	2019	271,228	166,667	89,254	33,471	560,620		
	2020	98,563	62,025	62,105	24,337	247,030		
Ratio 2020/2019 (%)		0.36	0.37	0.69	0.72	0.44	−103.20	0.000
Inpatient	2019	8,832	3,079	1,655	611	14,177		
	2020	4,317	1,529	1,129	580	7,555		
Ratio 2020/2019 (%)		0.48	0.49	0.68	0.94	0.53	−9.84	0.000

Mann-Whitney Test for grade variables.

*A p < 0.05 was considered statistically significant*.

**Figure 3:**
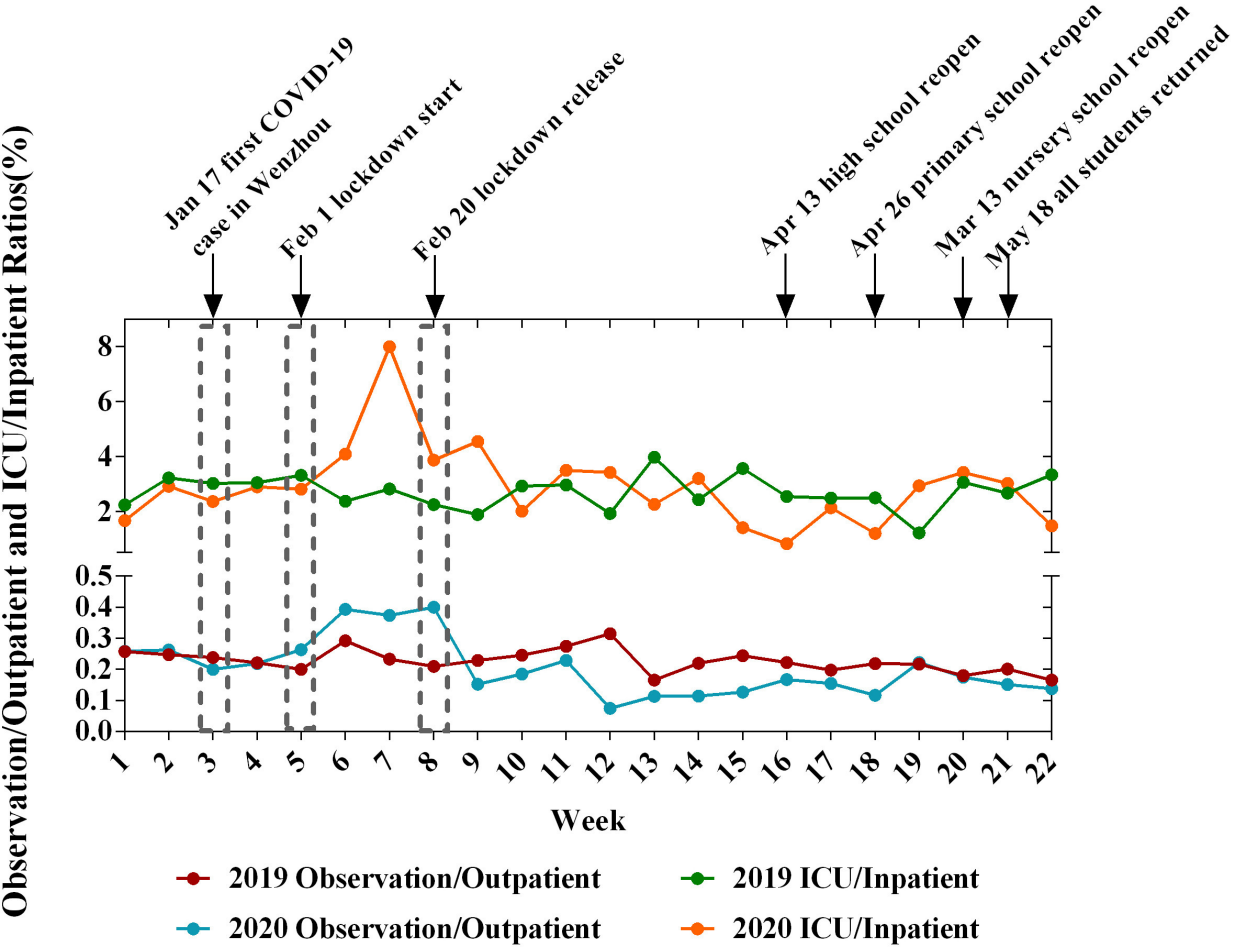
The ratio of EM observation/outpatient and ICU/inpatient during the 22-week period in 2019 and 2020.

According to the primary diagnosis, we classified all pediatric patients as having infectious or non-infectious diseases. During the COVID-19 outbreak, the proportion of infectious diseases in outpatients decreased significantly (*P* < 0.001), and a similar result was observed for inpatients (Table 1). Moreover, the number of patients decreased at the beginning of the epidemic, reached the lowest value during the lockdown period, and recovered after the lockdown. However, after lockdown, the trends of the infectious and non-infectious diseases exhibited a discernible difference (Figures 1, 2). Before the children returned to school, the number of patients showed a slow growth from a low baseline. The number of outpatients with non-infectious diseases in 2020 increased beginning in week 8, but the number of patients with infectious diseases did not increase until week 18 (Figure 1). Furthermore, both the ratios of emergency/outpatients and ICU/inpatients increased significantly during the lockdown period between weeks 5–8 (Figure 3).

## Discussion

The COVID-19 pandemic represents a major crisis challenging all aspects of healthcare systems worldwide (1, 10). The issue of child protection and pediatric clinic activity during the lockdown has been of particular concern (11). We reported the changes in Chinese pediatric departments in a specialized pediatric clinical center during the spread of the COVID-19 pandemic in different stages under different policies.

As the largest grade A class 3 children's hospital in southeast China, our hospital has an annual outpatient volume of over 1 million, with a wide range of sources and a complicated flow of patients. The number of patients requiring treatment at the pediatric department decreased significantly over a short period. The local government implemented multiple community containment measures on January 24, 2020, including quarantine, traffic control isolation, and social distancing, to prevent the spread of COVID-19 in Wenzhou (12). Quarantine is one of the effective tools for controlling communicable disease outbreaks, especially in virus disease pandemics (13). As a result of the “stay-at-home” order, the number of patient requests for outpatient clinic visits and wards decreased. In addition, the pandemic appeared to exert a greater effect on the treatment of younger children. Moreover, the ratios of emergency/outpatients and ICU/inpatients increased significantly during lockdown due to a delay of treatment or a decrease in the number of children with mild symptoms.

“Social distancing” is designed to reduce interactions among people in a broader community. In China, the government closed schools after the development of the epidemic to slow the spread of the infectious disease among children. According to previous studies, the main benefit of closing schools during an epidemic is to reduce transmission and new cases (14–16). Our study observed a decrease in the number of pediatric consultations after school closure, but with the reopening of schools, the number of visits gradually increased, particularly for infectious diseases. COVID-19 prompted a surge in the number of public policies adopted. In Wenzhou, the government strictly inspected the health standards for school resumption and implemented the students' registrations at different times. Before returning to school, the health information of teachers and students was obtained through the network. In addition, the school strengthened education about epidemic prevention between teachers and students through an online study. After resumption, the school continued to control the daily hygiene management measures on campus, such as applying health codes, temperature measurements, and daily disinfection. All of these policies effectively prevented the spread of the epidemic and slowed the spread of a number of infectious diseases. Furthermore, the need for tele-medicine services became more important to reduce the movement of patients (17). Therefore, doctors worldwide were encouraged to use the hospital cloud system to provide simple medical advice and treatment recommendations (18).

Children are the main population affected by infectious diseases, particularly acute respiratory infectious diseases (19). Acute respiratory tract infections (ARIs) are the main cause of morbidity among children aged <5 years in the developing world (20). Viruses cause up to 80% of ARIs (21), spreading via three different transmission routes: contact, droplet, and aerosol transmission (22). In 2019, infectious diseases were the main reason for pediatric outpatient and inpatient visits; however, the proportion of patients with infectious diseases decreased significantly in 2020 at our center, consistent with another recent study (11). A possible explanation is related to the reduced opportunity for cross-infection due to school closures and all children wearing masks. Moreover, the children were required to stay at home and received good care from their parents, resulting in a decrease in the number of pediatric patients presenting to the clinic. Based on our study, infectious diseases in children can be prevented and controlled. Prevention is the most important measure for preserving children's health (23).

A separate fever clinic was established in our hospital to screen children suspected of having COVID-19, preventing the spread of the epidemic. Outpatient preview and triage play important roles in the processing of outpatients with fever. Effective preview and triage can be used to screen and identify probable and suspected cases as early as possible (24). One-way entrances for the patients and designated staff paths were introduced at our hospital. In the medical ward, only one parent/caregiver was allowed to visit the patient, and this visitor was required to undergo screening at the hospital's screening booths before entry, leading to a decrease in the number of inpatients.

Some limitations should be considered when interpreting the findings of the present study. First, this study was a descriptive study, and our results might not be representative of other countries or regions. Second, the findings from the present study might not be transferable to other countries. COVID-19 may impact other healthcare systems differently in clinical practice in different countries. Third, our study employed a single-center design, which might affect its universal validity, particularly the criteria and the events requiring pediatric consultations and admission.

## Conclusion

We must be aware that the current crisis will have a significant impact on the pediatric healthcare system. There is an urgent need to improve pediatric infrastructure and improve staffing to manage the current pandemic and future pandemics. Pediatrics is not only facing new challenges, but also developmental opportunities, which requires us to constantly change our thinking and make necessary adjustments and adaptations to our practice.

## Data Availability Statement

All datasets generated for this study are included in the article/supplementary material.

## Ethics Statement

The studies involving human participants were reviewed and approved by the Ethics Committee of The Second Affiliated Hospital of Wenzhou Medical University. Written informed consent to participate in this study was provided by the participants' legal guardian/next of kin.

## Author Contributions

HZ, Z-KX, and W-XZ conceived the study. HZ, L-WG, Y-YG, and HY collected and analyzed the data. HZ and W-XZ wrote the paper. All authors approved the content of the manuscript.

## Conflict of Interest

The authors declare that the research was conducted in the absence of any commercial or financial relationships that could be construed as a potential conflict of interest.
